# The Effect of Artemisinin-Based Drugs vs Non-artemisinin-based Drugs on Gametophyte Carrying in the Body After the Treatment of Uncomplicated Falciparum Malaria: A Systematic Review and Meta-analysis

**DOI:** 10.3389/fphar.2021.707498

**Published:** 2022-01-06

**Authors:** Yuanyuan Zou, Nadia Julie, Shiyuan Guo, Yexiao Tang, Hongying Zhang, Zhiyong Xu, Wanting Wu, Yueming Yuan, Zhibin Wu, Wenfeng Guo, Changqing Li, Xinan Huang, Qin Xu, Changsheng Deng, Jianping Song, Qi Wang

**Affiliations:** ^1^ Artemisinin Research Center, Guangzhou University of Chinese Medicine, Guangzhou, China; ^2^ Institute of Science and Technology, Guangzhou University of Chinese Medicine, Guangzhou, China; ^3^ The First Affiliated Hospital of Guangzhou University of Chinese Medicine, Guangzhou, China; ^4^ Institute of Lung Diseases, Guangzhou Chest Hospital, Guangzhou, China

**Keywords:** artemisinin-based drugs, gametophyte, malaria, meta-analysis, review

## Abstract

The WHO recommends Artemisinin-based combination therapy (ACTs) as the first-line treatment for malaria. This meta-analysis aims to analyze the effects of artemisinin and its derivatives as well as non-artemisinin drugs on the gametophytes in the host during the treatment of falciparum malaria. Fourteen studies were included in this analysis, and the artemisinin combination drugs involved were: artemether-lumefantrine (AL), artemisinin (AST), artemether-benflumetol (AB), dihydroartemisinin-piperaquine + trimethoprim + primaquine (CV8), amodiaquine + sulfadoxine-pyrimethamine (ASP), pyronaridine-phosphate + dihydroartemisinin (PP-DHA), dihydroartemisinin (DHA), and mefloquine + artesunate (MA), with 1702 patients. The control intervention measures involved the following: sulfadoxine-pyrimethamine (SP), mefloquine (MQ), atovaquone-proguanil (AT-PG), chloroquine + sulfadoxine-pyrimethamine (C-SP), quinine (Q), pyronaridine-phosphate (PP), pyronaridine (PN), and mefloquine + primaquine (MP), with 833 patients. The effect of ACTs was more obvious (OR = 0.37, 95%CI: 0.22–0.62, *p* < 0.05). In the control group of second malaria attacks, the difference between the two groups was not statistically significant (RD = 1.16, 95%CI: 0.81–1.66, *p* < 0.05); there was no significant difference in treatment failure during follow-up (RD = -0.01, 95%CI: 0.04–0.03, *p* < 0.05). There were also very few serious adverse events in both groups. ACTs showed good therapeutic effects in preventing gametocythemia but did not control the recrudescence rate and overall cure, which indicated the effectiveness of the combination of antimalarial drugs. Further research is required to explore which compatibility method is most conducive to the development of clinical malaria control.

## Introduction

Malaria is one of the most important public health problems in the world. It has long threatened people’s health and life safety and affected social and economic development. In 2019, a total of 229 million cases of malaria were reported in 87 countries around the world, an increase of 1 million from 2018, to 409,000 deaths from malaria. Malaria is also a important human parasitic disease and a common cause of fever in tropical areas (Mehari et al., 2021). Its clinical manifestations include fever, anemia, and splenomegaly. There are five species of plasmodium that can infect humans: *Plasmodium falciparum* (P.f)—mainly causes severe malaria, *Plasmodium vivax*, *Plasmodium malariae*, *Plasmodium ovale*, and *Plasmodium knowlesi* (Desai et al., 2007; Lozano et al., 2010). The spread of malaria depends on the presence of gametophytes in peripheral blood (Johnston et al., 2014). After several generations of the proliferation of plasmodium in red blood cells, some merozoites enter the red blood cells to develop into gametophytes. After the Anopheles mosquito sucks blood, the mature gametophytes continue to complete the life cycle of malaria in the mosquitoes. The mechanism of formation of gametophytes is unclear and is hypothesized to be related to the density of infected asexual parasites, anemia, the duration of malaria symptoms, and the strength of immunity. Therefore, reducing the number and density of gametophytes in the host would significantly reduce the spread of malaria (Bousema and Drakeley, 2011; Essangui et al., 2019). At present, the elimination of malaria on a global scale is still facing huge challenges.

For countries in areas where malaria is endemic, the ultimate goal is not to cure the infected patients only once but to prevent recurrence or re-infection. With the emergence of resistance to antimalarial drugs, controlling malaria poses a considerable challenge. Some trials have proved that artemisinin combination therapy is effective in treating simple malaria and reducing the load of gametophytes in drug-resistant and non-drug-resistant lines (White, 1997; Durrani et al., 2005; Adjalley et al., 2011). In the past few years, large-scale deployment of insecticide-treated mosquito nets and effective artemisinin combination therapy (ACTs) to treat malaria has shown good results in malaria control (Bhatt et al., 2015). However, artemisinin, as a short-term high-efficiency single drug, often leads to a higher recurrence rate after treatment (Koram et al., 2005; Bukirwa et al., 2006), and ACTs have shown a little protective effect on malaria infection that relapses after treatment. Thus, it is necessary to focus on some intervention measures in the process of malaria transmission to better control or even eliminate malaria in the future. Therefore, while evaluating the transmission power of antimalarial drugs, in addition to the analysis of the gametophyte carrying criteria, the clinical treatment failure rate and recurrence rate are also very important (Griffin et al., 2010).

In this paper, we conducted a summary analysis of 14 experiments comparing artemisinin-based drugs and non-artemisinin-based antimalarial drugs. The primary endpoint is the rate of gametophyte carrying in the body after administration, and the secondary endpoint is the failure rate of clinical treatment failure and the rate of second malaria attacks during follow-up. Which may deepen the understanding mechanism of mediating malaria parasite human-mosquito-human transmission.

## Materials and Methods

### Study Strategy

In November 2020, we conducted a web-based systematic literature search in accordance with the PRISMA guidelines to determine all eligible clinical trials published between 1990 and 2020. The databases included MEDLINE, EMBASE, Web of Science, China National Knowledge Infrastructure (CNKI), Chinese Biomedical Database (CBM), and Cochrane Library. The search keywords were “uncomplicated falciparum malaria,” “gametophyte,” and “arteether OR dihydroartemisinin OR artemether OR artemisinin OR artesunate OR artemether-benflumetol OR artemether-lumefantrine.” We included trials that compared artemisinin combination drugs with non-artemisinin antimalarial drugs, and subsequent references were analyzed to find potentially eligible literature.

### Research Options

#### P:Patients

All patients infected with falciparum malaria without other complications were included in this study. There are no special requirements for age and gender. The inclusion criteria for patients were as follows: 1) Parasitemia ≥5000 *P falciparum*/µL; 2) Fever ≥37.5°C or a history of fever in the past 3 days; 3) The onset of malaria did not exceed 1 week. The exclusion criteria were as follows: 1) Patients with symptoms of severe malaria; 2) Pregnancy or breastfeeding patients; 3) Patients who had consumed antimalarial drugs and antibiotics in the past week or had an antimalarial history in the past 2 weeks; 4) Patients who had been infected with other malarial parasites; 5) Patients with severe malnutrition or a history of chronic diseases of the heart, liver, and kidney; 6) Patients with allergic reactions to antimalarial drugs.

#### I: Intervention

This study used ACTs as the main intervention measure, including artemether-lumefantrine (AL), artemisinin (AST), artemether-benflumetol (AB), dihydroartemisinin-piperaquine + trimethoprim + primaquine (CV8), amodiaquine + sulfadoxine-pyrimethamine (ASP), pyronaridine-phosphate + dihydroartemisinin (PP-DHA), dihydroartemisinin (DHA), and mefloquine + artesunate (MA). Although the drug brands used in the included studies were different, all drugs were manufactured by regular manufacturers with product numbers and were used in strict accordance with the recommended drug dosage.

#### C: Compare

The non-artemisinin antimalarial drugs (nACTs) were treated as the control group, including sulfadoxine-pyrimethamine (SP), mefloquine (MQ), atovaquone-proguanil (AT-PG), chloroquine + sulfadoxine-pyrimethamine (C -SP), quinine (Q), pyronaridine-phosphate (PP), pyronaridine (PN) and mefloquine + primaquine (MP). Similar to the intervention group, the dosage, and brand of the drugs were not required to be the same, but all drugs were manufactured by regular manufacturers with product numbers and were used in strict accordance with the recommended drug dosage.

#### O: Outcome

The transmission rate of falciparum malaria is known to be related to many factors. The primary endpoint of this study was the rate of patients with gametophytes after the completion of drug administration, and the secondary endpoints were the treatment failure rate and the rate of second malaria attacks. Gametophyte clearance was defined as follows: when under the microscope, no gametophyte (≥500 white blood cells) was found in the thick blood film. Clinical treatment failure was defined as follows: after the end of the treatment course, if the protozoan asexual body was still detected on the thick blood smear twice, the treatment was deemed invalid. The second episode of malaria was defined as follows: the second episode, including new infections and recurrences, i.e., the patient after the peripheral blood smear was identified as negative (no asexual plasmodium was found in the thick blood smear for two consecutive days (≥200 fields of view), and parasitemia was again observed under the microscope during the follow-up period.

#### S: Study

Randomized controlled trials (RCTs) evaluating ACTs and nACTs were included in this meta-analysis. We also included references, abstracts, and conference reports that provided relevant data.

### Data Management

Two independent examiners screened the titles, abstracts, and full texts generated by the electronic database search. Any differences of opinion arising during the period were resolved by mutual consensus. The reviewers collected the following information from each included study: first author, year of publication, age and characteristics of participants, confirmation of malaria, the dosage of experimental drugs used, the sample size of the intervention group and the comparison group, duration of treatment, the number of patients with gametophytes in their bodies, clinical cure rates, recurrence rates, adverse events, and follow-up time. “Clinical cure rates” was the 28-days parasitological cure rate. Which describes the proportion of patients with clearance of asexual parasitaemia within 7 days of initiating study treatment without recrudescence at day 28, based on blood smears. “Recurrence rate” refered to the proportion of the recurrence of asexual parasitaemia after treatment of the infection with the same infection that caused the original illness. This results from incomplete clearance of parasitaemia due to inadequate or ineffective treatment. It differs from a new infection or re-infection (as identified by molecular genotyping in endemic areas). If necessary, the original author was contacted for more information.

### Quality Assessment

The Cochrane System Deviation Risk Assessment Tool was used to assess the quality and deviation risk of the included clinical trials. The assessment tool specifically included the following seven aspects: 1) whether it was a generated random sequence; 2) whether it was randomly assigned; 3) whether the participants and personnel were double-blinded; 4) whether the result evaluation was blinded; 5) whether the result data were complete; 6) whether the report was selective; 7) whether there were other biases (Higgins et al., 2011). The quality of each study was classified as high risk, uncertain risk, or low risk. When the reviewer had any uncertainty about the research content of the report, the original author was contacted by e-mail or telephone. If the original author did not respond, then the study was excluded from this analysis.

### Statistical Analysis

The analysis results of the primary and secondary endpoints were expressed using relative risk and 95% confidence interval (CI). The χ^2^ test and the I^2^ test were used to evaluate the statistical heterogeneity between the combined experiments (Higgins et al., 2003). If I^2^ was 0–40%, it was classified as low heterogeneity, using the fixed-effects meta-analysis model, and if I^2^ was 40–100%, it was classified as high heterogeneity, using the random-effects meta-analysis model. The impact of the quality of the included studies on the results was evaluated through sensitivity analysis, and the evaluation of publication bias was evaluated through funnel plots. A *p*-value < 0.05 was considered as statistically significant. All data analysis was done using R Studio and Review Manager 5.3. This meta-analysis was registered with Prospero (registration number CRD42021229882).

## Results

### Search Results

**FIGURE 1:**
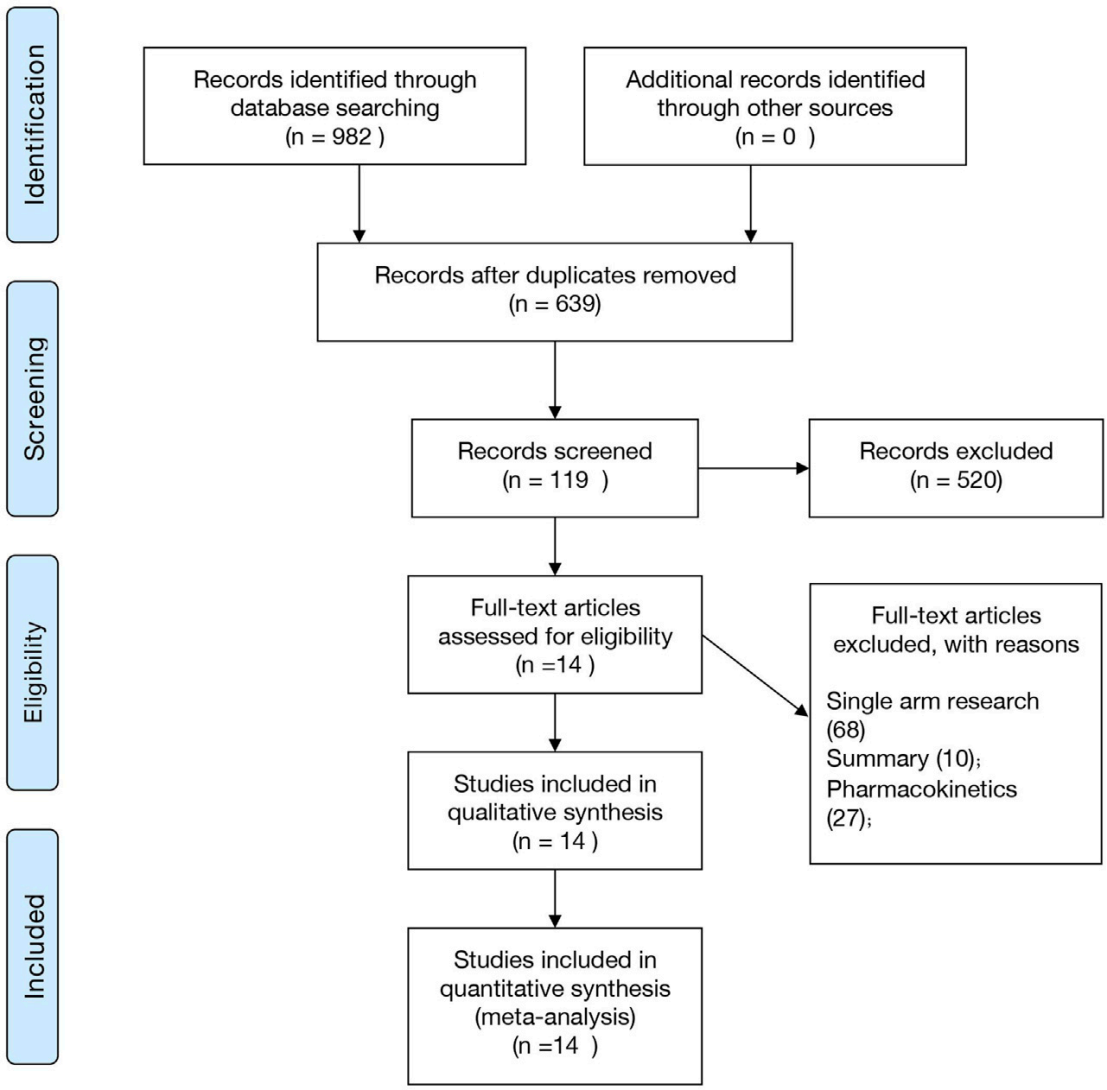
Retrieval selection process.

Figure 1 shows the retrieval selection process used in this study. During the initial search, 982 potential documents were identified, followed by the elimination of 343 repeated experiments. After the title review, 119 articles were included and the abstract and full text were further analyzed. Finally, we included 14 studies in this meta-analysis. Four studies were published in Chinese journals (Chen et al., 1994; Chen et al., 1999; Shang and Han, 2001; Liu et al., 2002), and ten studies were published in English-language journals (von Seidlein et al., 1998; Suputtamongkol et al., 2003; Giao et al., 2004; Mayxay et al., 2004; Bousema et al., 2006; Thapa et al., 2007; Zongo et al., 2007; Sowunmi et al., 2008; Achan et al., 2009; Okafor et al., 2010). Three of these studies compared AL and ASP (Zongo et al., 2007; Sowunmi et al., 2008; Okafor et al., 2010), and two compared AL and SP (Bousema et al., 2006; Thapa et al., 2007). The remaining nine studies compared AL and CSP (Mayxay et al., 2004), AST and MQ (Chen et al., 1994), AB and SP (von Seidlein et al., 1998), CV8 and AT-PG (Giao et al., 2004), AL and Q (Achan et al., 2009), PP-DHA and PP (Shang and Han, 2001), DHA and Q (Chen et al., 1999), MA and MP (Suputtamongkol et al., 2003), and DHA and PN (Liu et al., 2002). (See Supplementary Table S1).

### Study Characteristics

This meta-analysis included 14 RCTs. Table 1 shows the characteristics of each trial. Among these RCTs, two studies were conducted in China (Chen et al., 1994; Liu et al., 2002), two were conducted in Thailand (von Seidlein et al., 1998; Suputtamongkol et al., 2003), two were conducted in Vietnam (Chen et al., 1999; Giao et al., 2004), and the rest were conducted in Kenya (Bousema et al., 2006), Nepal (Thapa et al., 2007), Nigeria (Okafor et al., 2010), Burkina (Zongo et al., 2007), Laos (Mayxay et al., 2004), Uganda (Achan et al., 2009), Ibadan (Sowunmi et al., 2008), and Africa (Shang and Han, 2001). In this analysis, we report the results related to the use of ACTs and nACTs for the treatment of uncomplicated falciparum malaria. All included studies evaluated the changes in the body’s gametophytes during treatment, except for two studies (Mayxay et al., 2004; Thapa et al., 2007). These two studies provided data on changes in the density of gametophytes before and after treatment, while the remaining 12 studies provided the number of patients with gametophytes detected by microscopic blood tests. These studies enrolled 2,535 patients and were published between 1990 and 2020. The experimental group included 1702 patients who received ACTs and 833 patients who received nACTs. A written informed consent had been collected from the participating patients and their guardians, and these experiments had been approved by the local ethics committee. Moreover, the authors of these 14 studies stated that there was no conflict of interest between the manufacturers of the drugs used in the experiments, and the drug manufacturers did not participate in the design and implementation of the trial protocol.

**TABLE 1:** Characteristics of the included studies.

Author	Country	Study population	N(F)	Comparators (number)	Course of treatment (Days)	PCT(h)	FCT(h)	Adverse reactions(N)
Chen et al., 1994	China	NR	18(NR)	AST (9)	5	NR	NR	NR
				MQ (9)	5			
von Seidlein et al., 1998	Thailand	children	287(NR)	AB (144)	4	NR	NR	NR
				SP(143)	4			
Chen et al. (1999)	Vietnam	>15 years old	18(NR)	DHA (10)	5	54.6 ± 22.4	NR	NR
				Q (8)	7	109.3 ± 46.2		
Shang and Han (2001)	Africa	adults + children	24(NR)	PP-DHA (13)	2	27.69	NR	NR
				PP(11)	3	50.18		
Liu et al. (2002)	China	adults + children	49(NR)	DHA (24)	7	NR	NR	NR
				PN(25)	3			
Suputtamongkol et al. (2003)	Thailand	adults + children	418(NR)	MA (238)	3	NR	NR	NR
				MP(177)	3			
Giao et al. (2004)	Vietnam	adults	161 (145)	CV8(82)	3	34.8 (30.9–38.6)	24.6 (22.3–26.8)	Itching (1)headache (1)
				AT-PG (79)	3	34.5 (30.7–38.2)	23.5 (20.8–26.2)	Itching (1)
Mayxay et al. (2004)	Laos	>1 years old	220 (88)	AL (110)	3	41.6 (48–50.4)	23 (20.9–25.3)	Abdominal pain (2) Headache (12) Skin rash (2) Vomiting (1)
				C-SP(110)	3	69.6 (67.2–72)	40.2 (35.9–44.4)	Abdominal pain (7) Headache (12) Skin rash (0) Vomiting (8)
Bousema et al. (2006)	Kenyan	children	227 (108)	AL (75)	3	NR	NR	NR
				SP(152)	3			
Thapa et al. (2007)	Nepal	>5 years old	99 (39)	AL (66)	3	31 ± 16	24 (12–60)	Headache (32) Vomiting (13)
				SP(33)	3	67 ± 32	48 (18–72)	Headache (58) Vomiting (30)
Zongo et al. (2007)	Burkina	>6 months	521 (276)	AL (261)	3	NR	NR	Abdominal pain (11) Headache (10)
				ASP(142)	3			Abdominal pain (18) headache (14)
Sowunmi et al. (2008)	Ibadan	children	181 (98)	AL (98)	3	NR	NR	Itching (0)
				ASP(91)	3			Itching (2)
Achan et al. (2009)	Uganda	6–59 months	175 (94)	AL (89)	7	NR	NR	Vomiting (2) Diarrhea (3) Skin rash (0)
				Q (86)	7			Vomiting (1) Diarrhea (0) Skin rash (1)
Okafor et al. (2010)	Nigeria	children	140(NR)	AL (70)	3	NR	NR	Abdominal discomfort (10) Rash (2)
				ASP(70)	3			Abdominal discomfort (8) Rash (0)

NR:not report; AST: artemisinin; MQ: melfloquine; AL; artemether-lumefantrine; SP: sulfadoxine-pyrimethamine; AB: artemether-benflumetol; CV8:dihydroartemisinin-piperaquine + trimethoprim + primaquine; AT-PG:atovaquone-proguanil; ASP: amodiaquine + sulfadoxine-pyrimethamine; C-SP: chloroquine + sulfadoxine-pyrimethamine; Q:quinine; PP-DHA:pyronaridine-phosphate + dihydroartemisinin; DHA:dihydroartemisinin; PP: pyronaridine-phosphate; PN: pyronaridine; MA: melfloquine + artesunate; MP: melfloquine + primaquine.

### Trial Quality

The Cochrane bias risk assessment tool was used to evaluate the methodological quality of the included studies. The evaluation method included a comprehensive evaluation of the following seven aspects: 1) whether the random sequence was generated; 2) whether the allocation was hidden; 3) whether the result evaluation was blinded; 4) whether the patients and researchers were blinded; 5) whether the incomplete result data was processed; 6) whether the data was deliberately selected for reporting; 7) whether other potential biases existed. All evaluations were derived from the original text of each study. For the original text without a clear description, we evaluated it as low-risk or unclear based on the content of the full text. Based on these seven evaluation criteria, five or more studies were found to be low risk. We classified them as “low risk of bias” trials. The 12 experimental studies included were all low risk of bias (Table 2).

**TABLE 2:** Bias and risks of included studies.

Description of domains	Author, publication year													
	Chen et al. (1994)	von Seidlein et al. (1998)	Chen et al. (1999)	Shang and Han (2001)	Liu et al. (2002)	Suputtamongkol et al. (2003)	Giao et al. (2004)	Mayxay et al. (2004)	Bousema et al. (2006)	Thapa et al. (2007)	Zongo et al. (2007)	Sowunmi et al. (2008)	Achan et al. (2009)	Okafor et al. (2010)
Random sequence generation	yes	yes	Yes	yes	yes	yes	Yes	yes	yes	Yes	yes	yes	yes	yes
Allocation concealment	yes	yes	Yes	yes	yes	yes	Yes	yes	yes	yes	yes	yes	yes	yes
Blinding of outcome assessment	yes	yes	unclear	yes	unclear	yes	yes	unclear	yes	yes	unclear	yes	yes	yes
Blinding of participants and personnel	unclear	no	No	unclear	yes	no	unclear	yes	yes	no	yea	unclear	no	yes
Incomplete outcome data adequately addressed	yes	yes	yes	yes	yes	yes	yes	yes	yes	yes	yes	yes	yes	yes
Free of selecting outcome reporting	yes	yes	yes	yes	yes	yes	yes	yes	yes	yes	yes	yes	yes	yes
Other sources of potential bias	unclear	yes	unclear	unclear	unclear	yes	unclear	yes	unclear	Unclear	Unclear	unclear	unclear	unclear

Yes = low risk of bias; Unclear = uncertain risk of bias; No = high risk of bias.

### Primary Endpoint—Gametophyte Carrying in the Patient

**FIGURE 2:**
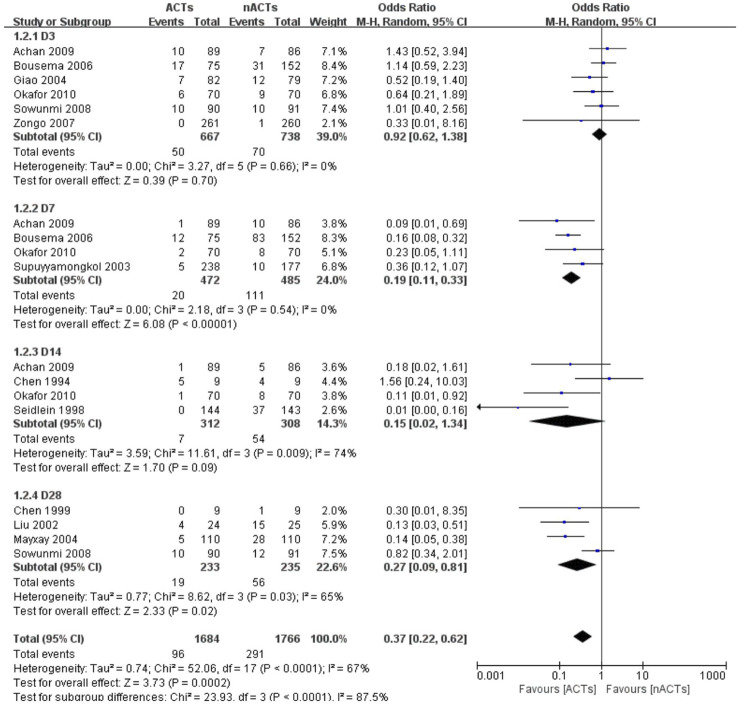
Gametophyte carrying in the patient.

The authors claimed that there was no statistical difference between the baseline and the distribution of each group for all patients included in the study before receiving treatment. Except for one study, there was a huge disparity in the number of people receiving ACTs and nACTs, i.e., 66 patients received AL treatment, and 33 participants received SP treatment, but the author believed that this difference was acceptable (Thapa et al., 2007). Since the course of treatment in each study was not the same, and the set follow-up time was not the same. We selected the data on the third day (Giao et al., 2004; Bousema et al., 2006; Zongo et al., 2007; Sowunmi et al., 2008; Achan et al., 2009; Okafor et al., 2010), the seventh day (Shang and Han, 2001; Suputtamongkol et al., 2003; Bousema et al., 2006; Achan et al., 2009), the 14th day (Chen et al., 1999; Okafor et al., 2010; von Seidlein et al., 1998) and the 28th day (Chen et al., 1999; Zongo et al., 2007; Sowunmi et al., 2008) for subgroup and overall analysis. One of the studies only gave the gametophyte appearance rate and did not indicate that it was in the first few days of follow-up (Liu et al., 2002).^14^ Since the follow-up period of this study was 28 days. We divided it into the 28th day gamete appearance rate. Based on the analysis results, we found that on the third day of treatment, the difference between ACTs and nACTs was not statistically significant (OR: 0.92, 95% CI: 0.62–1.38). However, after 1 week of treatment, there was a significant difference in the gametophyte carrying between the two groups of patients (OR: 0.19, 95% CI: 0.11–0.33). At the second week of follow-up, the difference between ACTs and nACTs became insignificant (OR: 0.15, 95% CI: 0.02–1.34). On the 28th day of follow-up, there was a statistically significant difference between ACTs and nACTs (OR: 0.27, 95% CI: 0.29–0.81). Thus, we found that there was a statistically significant difference between ACTs and nACTs (OR: 0.37, 95% CI: 0.22–0.62). (Figure 2).

### Research Publication Bias and Sensitivity Analyses

We use funnel plots to determine the publication bias of the primary endpoint data. There was one study on the left side of the funnel chart and two studies on the right side (Figure.5.1–A). Since the credibility interval of the random-effects model was wider than that of the fixed-effects model, the random-effects model was used for analysis, and there were statistical differences between these studies (OR: 0.2581, 95%CI: 0.1784–0.3736, *p* < 0.001). After correction, the overall effect size was still statistically significant; thus, the possibility of publication bias was ruled out. Sensitivity analysis was conducted on 11 studies that reported the rate of gametophyte carrying conditions after 7 days of treatment, and the results showed that good stability of the analysis results (OR: 0.2581, 95%CI: 0.1784–0.3736, *p* < 0.001) (Figures 5.1–B).

### Secondary Endpoint 1—Clinical Treatment Failure Rate

**FIGURE 3:**
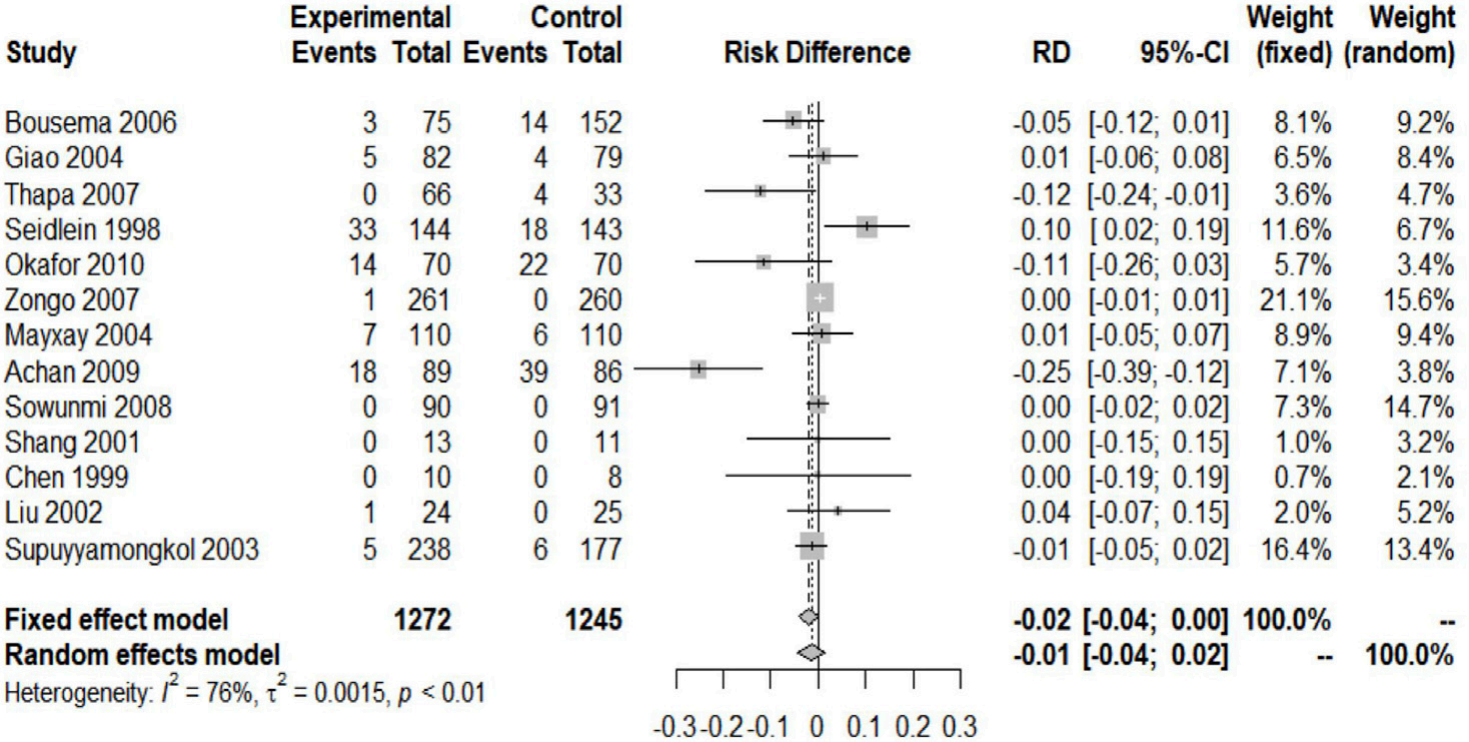
Clinical treatment failure rate.

Except for one study that did not report the clinical cure rate, others included experiments reporting the relevant data (Chen et al., 1994). We included 2517 patients in the analysis of the failure rate of clinical treatment. Since I^2^ = 76% > 40%, a random-effects model was used, and the analysis result obtained showed the absence of significant difference between the two groups of ACTs and nACTs in the efficacy of curing patients (RD: 0.01, 95% CI: 0.04–0.02, *p* < 0.01). (Figure 3).

### Research Publication Bias and Sensitivity Analyses

There were one and two studies on the left and right sides of the figure, respectively (Figures 5.2–A). Similarly, the CI of the random-effects model was wider than that of the fixed-effects model; thus, the random-effects model was used (RD: 0.0028, 95%CI: 0.0254–0.0309, *p* > 0.05). The results showed the absence of a statistically significant difference. In the sensitivity analysis (Figures 5.2–B), we found that the results of one study were unstable (von Seidlein et al., 1998) (t = 0.054 > 0.05). This was an intention-to-treat study. In fact, 144 participants were included in the ACTs group, of which 119 patients were evaluated, of which 111 were cured. Also, 143 people were included in the nACTs group, of which 128 were evaluated, of which 125 were cured. The rest of the research and analysis results were stable.

### Secondary Endpoint 2 — Second Malaria Attack Rate

**FIGURE 4:**
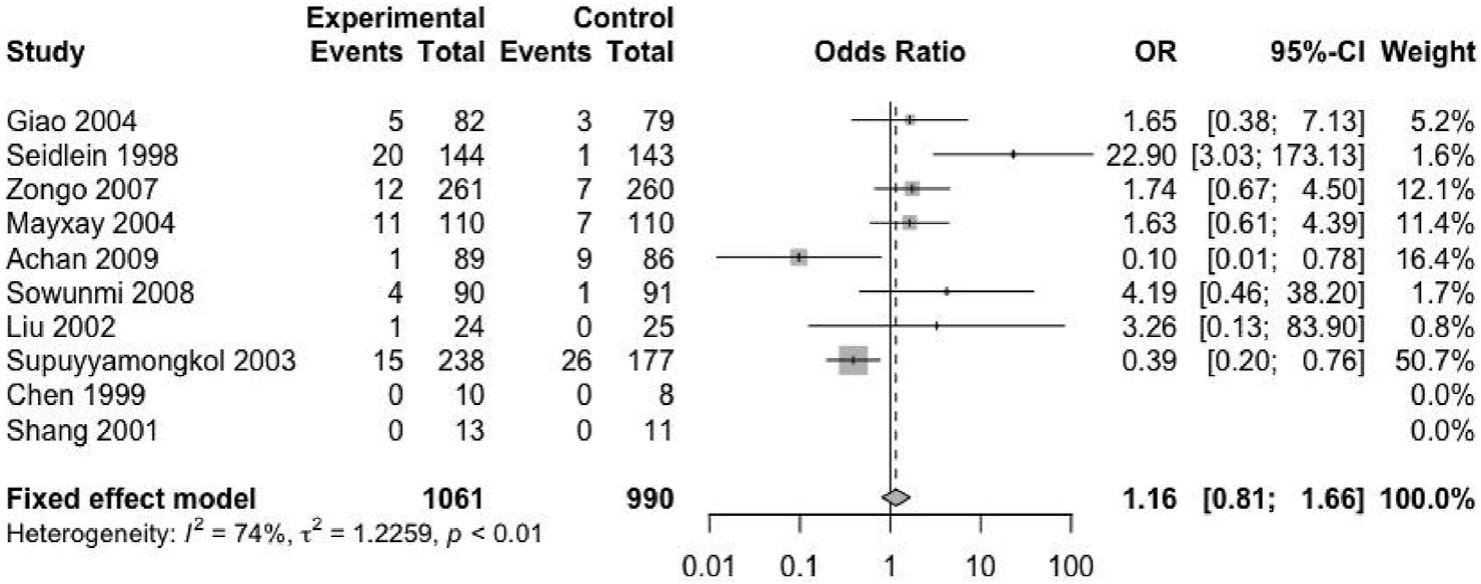
Second malaria attack rate.

Except for four included trials, no second episode (including new infection and recurrence) was reported (Chen et al., 1994; Bousema et al., 2006; Thapa et al., 2007; Okafor et al., 2010). The remaining trials provided the relevant data. The studies reported 2051 patients with malaria recurrence, of which 1,061 received ACTs and 990 received nACTs. Due to the high heterogeneity (I^2^ = 74%), we adopted a random-effects model, and the results showed that there was no significant difference in the recurrence rate between the two groups (RD: 1.16, 95% CI: 0.81–1.66, *p* < 0.01) (Figure 4).

### Research Publication Bias and Sensitivity Analyses

There were two studies and one study on the left and right sides of the funnel chart, respectively (Figures 5.3–A). The confidence interval of the fixed-effects model was narrower than that of the random effects-model; thus, we used a random-effects model for analysis, and the results showed no statistical significance (RD: 0.7805, 95%CI: 0.3027–2.0127, *p* = 0.6082 > 0.05). When analyzing the sensitivity (Figure.5.3-B), we found that the results of one study were not stable (Suputtamongkol et al., 2003). This study was done in western Thailand, and the resistance of this mefloquine was very high; thus, the data results were not stable. The rest of the tests have good stability.

**FIGURE 5:**
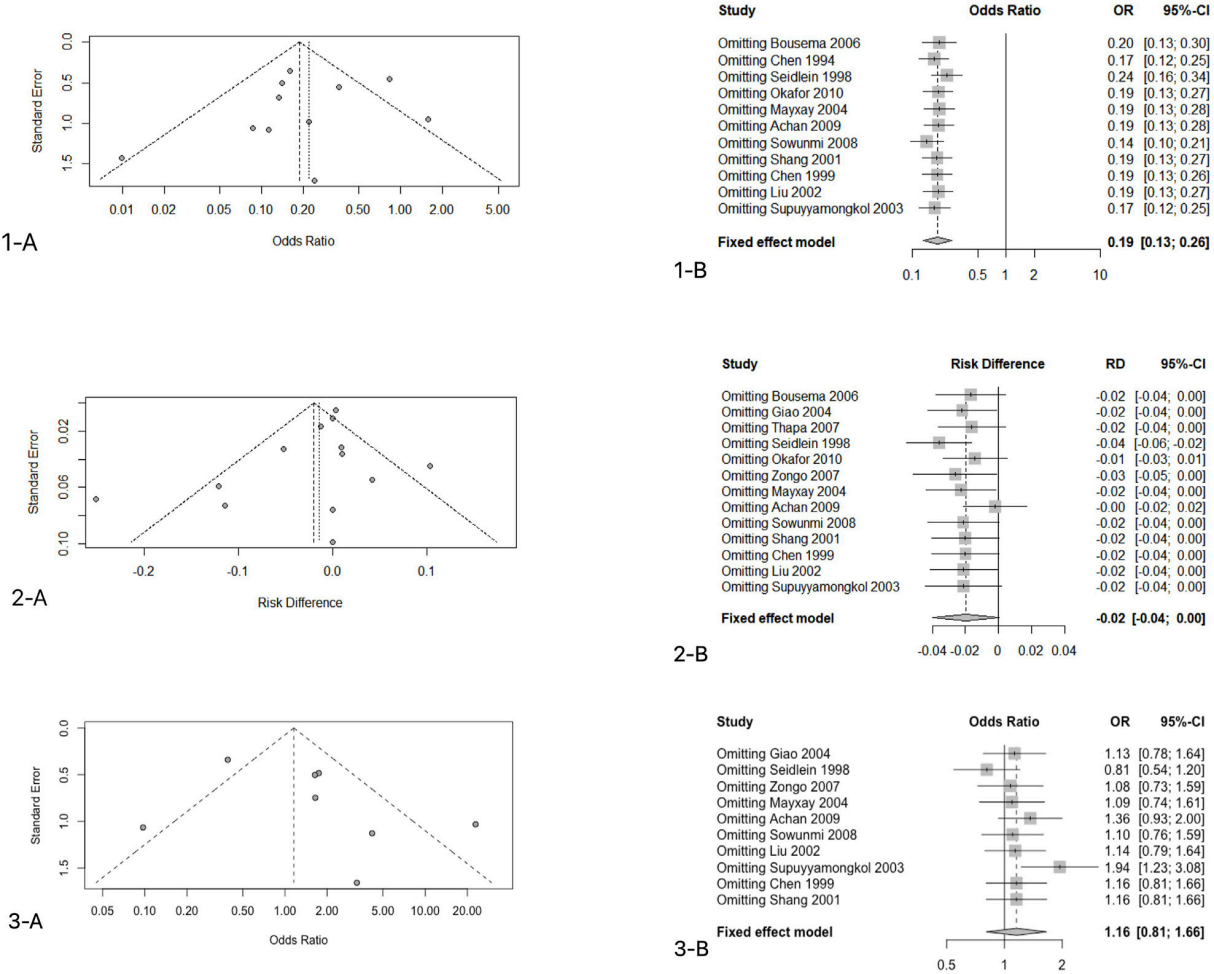
Research publication bias and sensitivity analyses.

## Discussion

Generally, antimalarial drugs target the asexual blood stage of *P. falciparum*, which mainly causes clinical disease and death in patients. While the parasites in the sexual stage, gametophytes, do not cause clinical diseases, they are found in the blood circulation of patients and infect mosquitoes, which grow in the mosquitoes and then infect humans. In many areas south of the Sahara, it has been reported that *P. falciparum* has developed resistance to multiple antimalarial drugs (Attaran et al., 2004), due to which more and more people receive combination therapy instead of a single drug. Artemisinin and its derivatives are the first antimalarial drugs (Yeung et al., 2004), which have the characteristics of fast onset, wide distribution, rapid excretion, and high recurrence rate.

The mature gametophyte of *P. falciparum* first appears in human blood after being infected with malaria for 7–15 days. Artemisinin and its derivatives, such as artemether, etc., are converted into dihydroartemisinin in the human body (Yan et al., 2019). It quickly eliminates the malaria parasite and reduces fever in the patient’s body. The asexual phase of the malaria parasite has a longer active period than other quinoline or antifolate drugs, from the ring phase to the early trophozoite artemisinin, and rapidly produces therapeutic effects. Its rapid onset characteristics inhibit the further development of severe malaria and partially delay the development of drug resistance. ACT possesses the ability to kill the developing gametophytes (Kumar and Zheng, 1990) or cause male gametophytes to stay in deep tissues instead of being released into the body’s peripheral blood circulation (Paul et al., 2000). When combined with a suitable compatible drug, its drug absorption rate increases, which can be maintained in the body’s blood for 3 days. It also delays the resistance of the compatible drug and reduces the number of gametophytes, and the compatible drug also delays the development of resistance against artemisinin. When artemisinin is metabolized in the body, compatible drugs are used to remove the residual protozoa in the blood (which reduces the density by hundreds of millions of times even before receiving treatment).

We included 2535 patients for meta-analysis, of which 1702 received ACTs: 761 received AL, 9 received AST, 144 received AB, 82 received CV8, 421 received ASP, 13 received PP-DHA, 34 received DHA, and 238 received MA. Among the 833 people who received nACTs: 25 received PN, 17 received MP, 79 received AT-PG, 328 received SP, 9 received MQ, 110 received C-SP, 94 received Q, and 11 received PP. The results of these studies showed that in the first 4 weeks of treatment, the gametocytes of patients treated with ACTs were significantly lower in the nACTs group.

SP has a long half-life; it mainly interferes with the folic acid synthesis pathway of the malaria parasite by inhibiting the synthesis of biological enzymes, such as dihydrofolate synthesis reductase-thymidylate synthase, thereby exhibiting a pesticidal effect. SP inhibits the maturation of trophozoites of asexual malaria parasites and also acts on the prophase of red blood cells to kill sporozoites. Moreover, it has a good curative effect, is cost effective, and has high availability. However, the use of drugs with a long half-life increases the risk of parasite resistance (Ochong et al., 2003). Several years after SP entered clinical trials, it was reported that *P. falciparum* and *P. vivax* developed resistance to it (Abdul-Ghani et al., 2013).

The half-life of L is similar to that of SP; it has a short treatment course and is well tolerated when combined with artemether. Fourteen days of treatment results in a significant reduction in the proportion of infected mosquitoes, which partially inhibits the spread of malaria. However, the drug requires higher fat content in meals; thus, it is costly with a lower rate of patient compliance.

Chloroquine (C) is a 4-aminoquinoline drug that mainly acts on the large and small loops and trophozoites of mature malaria parasites. It has a heat-clearing effect, and the activity has early specificity. Although the protozoan clearance rate is slower than that of artemisinin derivatives, it is well-tolerated, and its combination with SP significantly improves the fever clearance time and protozoan clearance time. Also, a study found that the initial response of C-SP was significantly better than that of AL (Mayxay et al., 2003). However, due to the emergence of recurrent resistance to Plasmodium falciparum, some areas had to stop using chloroquine and sulfadiazine as antimalarial drugs (Mekonnen et al., 2014; Madkhali et al., 2021).

Amodiaquine (A) combined with SP has been found to be more effective than AL in reducing the overall incidence of malaria after treatment. Also, AL has a little protective effect on malaria recurrence after treatment, while ASP has a good protective effect on new infections (Zongo et al., 2007).

Quinine (Q) is the first proven antimalarial drug and is administered for approximately 7 days. It is well known that it has poor tolerance and poor compliance (Achan et al., 2011), and a short course of treatment or low patient compliance has been shown to significantly reduce its efficacy (Sanchez et al., 2016). It mainly acts on the mature trophozoite stage of the protozoan development; however, it cannot prevent the adhesion of the protozoa and has little effect on the developed schizont.

Primaquine (PQ) is an 8-aminoquinoline drug with antimalarial activity similar to other 4-aminoquinoline drugs. It acts on the dormant seeds of *P. vivax* to prevent recurrence and acts on the gametophytes of *P. falciparum* to prevent its spread. It inhibits all activities of Plasmodium in the liver stage, which is the primary basis of preventive drugs. PQ interferes with heme polymerization (Parapini et al., 2000), and combined with DHA plus trimethoprim, it formed a new drug CV8, which could improve the short half-life of DHA by extending it to 17–25 days (Hung et al., 2003).

AT-PG is also effective against multi-drug resistant *P. falciparum* (Bustos et al., 1999). AT is a very effective antimalarial drug, but when used alone, it rapidly develops drug resistance. However, the bioavailability of the components of AT-PG is poor. It is expensive, and thus, not suitable for wide application (Hudson et al., 1991).

Benflumetol (B) is a relatively slow-acting anti-schizophrenia drug with a half-life of 4–6 days (Na-Bangchang et al., 1999a). Long half-life and long action time can prevent a recurrence, but the insecticidal speed is slow, and thus, the control of clinical symptoms is also relatively slow. When combined with artemether, which has a short half-life and acts on the erythroid phase, it can be effective against several drug-resistant malaria parasites. It is well tolerated and can effectively reduce the spread of malaria (Na-Bangchang et al., 1999b).

The mechanism of action of mefloquine (MQ) is similar to that of Q. It acts on falciparum malaria with multidrug resistance. It is an effective plasmodium asexual killer but has no direct effect on gametophytes. The gametophyte carrying rate is high after a large single dose (Price et al., 1996; Rufener et al., 2018). Although the combination of MQ and artesunate cannot kill the mature gametophyte, it prevents the development of the gametophyte through the precursor of the sexual stage and thus, improves the tolerance of MQ to partially speed up the recovery of the patients (Ferreira et al., 2018).

Pyronaridine (PN) is a highly effective and low-toxicity red endogenous schizont killer. It has a short curative effect with a low re-combustion rate after treatment, but it has no effect on the gametophytes in the body, and the time to clear the patient’s fever is slightly longer. Also, the gametophyte rate after single drug use is high (Yan et al., 2019). Pyronaridine-phosphate (PP) has a killing effect on the schizonts of *P. falciparum*.

Here, we divided the treatment methods into ACTs and nACTs. In terms of the overall cure rate, since we adopted an intention-to-treat method, the loss of people involved in the evaluation during the follow-up period was relatively large, which had a relatively large impact on the overall results (von Seidlein et al., 1998). Additionally, for the long-term studies, it was difficult to distinguish whether the recurrence of clinical symptoms of malaria during the follow-up period was due to new infection or a recurrence; thus, we could only assess the overall risk of recurrence. Therefore, the stability of the analysis results of the secondary endpoint was not very good. Moreover, some research sites were in areas with high malaria transmission, which also implied that they were challenged by more frequent new infections. As a short-acting antimalarial drug, artemisinin did not show significant superiority in preventing secondary attacks during the follow-up period.

However, the results of the primary endpoint showed that, compared with previous non-artemisinin-based first-line antimalarials, ACTs could significantly reduce the body’s gametocytes, although they could not completely prevent the second malaria attack. As more and more areas use ACTs as the primary antimalarial drug, and families have adopted a variety of defensive measures, such as using soaked mosquito nets to eliminate nearby mosquito breeding sites, etc., the transmission capacity of malaria might be effectively controlled.

## Conclusion

As a parasitic infectious disease, malaria has affected humans for centuries. Although the cause of the disease might be insignificant, the consequent problem is big. About half of the world’s population is at risk of developing malaria, and most of these infections occur in sub-Saharan Africa. With the development of drug resistance and considering the economic impact, antimalarial drugs have gradually changed from nACTs to ACTs.

We systematically analyzed the effects of ACTs and nACTs on host gametophytes during the treatment of falciparum malaria, the failure rate of clinical treatment, and the rate of secondary malaria attacks, which are considered essential for judging the efficacy of drugs. The analysis results showed that compared with the previous non-artemisinin first-line antimalarial drugs, although ACTs did not show a better trend in preventing secondary attacks and clinical cure rates. But ACTs could significantly reduce the body’s gametocytosis. It is a work which deepened the understanding mechanism of mediating malaria parasite human-mosquito-human transmission, and offered great potential for new targeted interventions to block parasite transmission from humans to mosquitoes to aid the elimination of malaria. And it was essential for controlling the further spread of malaria and reducing the overall incidence of an area.

### Study Limitations

Influencing factors on the transmission of malaria are not only related to the carrying density and time of gametophytes in the body but also to the sex ratio of gametophytes in the peripheral blood and the infectivity of protozoa to mosquitoes (Tchuinkam et al., 1993). Therefore, in the follow-up evaluation of the effectiveness of antimalarial compound drugs, apart from the basic FCT, PCT, gamete carrying rate, and recurrence rate were also recorded. The impact on the sex ratio of gametophytes is also worth investigating, which might be of great significance for controlling the spread of malaria. More clinical trials of antimalarial drugs with good efficacy, low price, good compliance and good tolerance are needed. Future research should also measure the infectivity of carriers of submicroscopic density gametophytes to further clarify the contribution of these individuals in transmission post-treatment.

## Data Availability

The original contributions presented in the study are included in the article/Supplementary Material, further inquiries can be directed to the corresponding authors.
