# Supplementary material for: The Effect of Artemisinin-Based Drugs vs Non-artemisinin-based Drugs on Gametophyte Carrying in the Body After the Treatment of Uncomplicated Falciparum Malaria: A Systematic Review and Meta-analysis
Source: Front Pharmacol. 2022 Jan 6;12:707498. doi: 10.3389/fphar.2021.707498 (PMC8770988; doi:10.3389/fphar.2021.707498)
Supplement: Supplementary file 1 [file Table1.docx]

Supplementary 1: The drug control table included in the study and its abbreviations in this article.

| **Author (year)** | **ACT** | **Abbreviations in this article** | **nACTs** | **Abbreviations in this article** |
| --- | --- | --- | --- | --- |
| Chen 1994 | artemisinin | AST | mefloquine | MQ |
| Seidlein 1997 | artemether-benflumetol | AB | sulfadoxine-pyrimethamine | SP |
| Chen 1999 | dihydroartemisinin | DHA | quinine | Q |
| Shang 2001 | pyronaridine-phosphate + dihydroartemisinin | PP-DHA | pyronaridine-phosphate | PP |
| Liu 2002 | dihydroartemisinin | DHA | pyronaridine | PN |
| Supuyyamongkol 2003 | mefloquine + artesunate | MA | mefloquine + primaquine | MP |
| Giao 2004 | dihydroartemisinin-piperaquine + trimethoprim + primaquine | CV8 | atovaquone-proguanil | AT-PG |
| Mayxay 2004 | artemether-lumefantrine | AL | chloroquine + sulfadoxine-pyrimethamine | C-SP |
| Bousema 2006 | artemether-lumefantrine | AL | sulfadoxine-pyrimethamine | SP |
| Thapa 2007 | artemether-lumefantrine | AL | sulfadoxine-pyrimethamine | SP |
| Zongo 2007 | artemether-lumefantrine | AL | amodiaquine + sulfadoxine-pyrimethamine | ASP |
| Sowunmi 2008 | artemether-lumefantrine | AL | amodiaquine + sulfadoxine-pyrimethamine | ASP |
| Achan 2009 | artemether-lumefantrine | AL | quinine | Q |
| Okafor 2010 | artemether-lumefantrine | AL | amodiaquine + sulfadoxine-pyrimethamine | ASP |
